# Draft Genome Sequence of *Tatumella* sp. Strain UCD-D_suzukii (Phylum *Proteobacteria*) Isolated from *Drosophila suzukii* Larvae

**DOI:** 10.1128/genomeA.00349-14

**Published:** 2014-04-24

**Authors:** Madison I. Dunitz, Pamela M. James, Guillaume Jospin, Jonathan A. Eisen, David A. Coil, James Angus Chandler

**Affiliations:** a University of California Davis Genome Center, Davis, California, USA; b Department of Evolution and Ecology, University of California Davis, Davis, California, USA; c Department of Medical Microbiology and Immunology, University of California Davis, Davis, California, USA

## Abstract

Here we present the draft genome of *Tatumella* sp. strain UCD-D_suzukii, the first member of this genus to be sequenced. The genome contains 3,602,931 bp in 72 scaffolds. This strain was isolated from *Drosophila suzukii* larvae as part of a larger project to study the microbiota of *D. suzukii*.

## GENOME ANNOUNCEMENT

*Tatumella* sp. strain UCD-D_suzukii was isolated from *Drosophila suzukii* larvae collected at the Wolfskill Experimental Orchard near Winters, CA. On 28 June 2012 undamaged whole cherries were collected in sterile plastic bags for transport to the University of California, Davis. The cherries were macerated in the bags and the largest visible larvae were picked from the bags, externally washed in 70% ethanol, rinsed in sterile water, and then individually placed in yeast extract-peptone-dextrose (YEPD) plates (1% yeast extract, 2% peptone, and 2% glucose/dextrose) (methods adapted from [1]). The larvae were allowed to migrate for 30 to 60 s and then transferred to a vial containing Bloomington *Drosophila* medium. All eclosing adults were identified as *D. suzukii*.

The resulting colonies were double-dilution streaked onto YEPD plates and incubated for 5 days at 20°C. Single colonies were transferred and incubated for 48 h in liquid YEPD medium at 20°C. Genomic DNA was extracted using a Wizard genomic DNA purification kit (Promega) from fresh overnight cultures. Illumina paired-end libraries were then made from sonicated DNA using a TruSeq DNA sample prep v2 kit (Illumina).

A total of 4,503,930 paired-end reads were generated on an Illumina MiSeq, at a read length of 300 bp. Quality trimming and error correction resulted in 3,975,054 high-quality reads. All sequence processing and assembly were performed using the A5 assembly pipeline (2). The assembly produced 85 contigs, contained in 72 scaffolds (minimum, 598 bp; maximum, 441,247 bp; *N*_50_, 329,990 bp). The final assembly contained 3,602,931 bp with a GC content of 51.44, and estimated overall coverage of 330×. Completeness of the genome was assessed using Phylosift (3), which searches for 37 highly conserved, single-copy marker genes (4), all of which were found in this assembly.

Automated annotation was performed using the RAST server (5). *Tatumella* sp. strain UCD-D_suzukii contains 3,725 predicted coding sequences and 110 predicted RNAs. A full-length (1,499 bp) 16S sequence was obtained from this annotation and was used to attempt to identify the species of *Tatumella*. This sequence is 100% identical to the representative sequence of the most common *Tatumella* operational taxonomic unit (OTU) found with *D. suzukii* larvae (J. A. Chandler, P. James, G. Jospin, and J. Lang, submitted for publication). Due to the recent transfer of select *Pantoea* into the *Tatumella* genus (6), the 16 s sequence was aligned with sequences from both genera in RDP (7), this alignment was then used to construct a phylogenetic tree in RAxML (8). *Tatumella* sp. strain UCD-D_suzukii is most closely related to *Tatumella ptyseos*, but is more than 99% identical to at least one other *Tatumella* species; therefore, we are unable to assign a species name to this isolate. Multiple *Pantoea* genomes have been published; however, none belong to the three species that were transferred to the *Tatumella* genus, making *Tatumella* sp. strain UCD-D_suzukii the first *Tatumella* genome to be sequenced.

### Nucleotide sequence accession numbers.

This whole-genome shotgun project has been deposited at DDBJ/EMBL/GenBank under the accession number JFJX00000000. The version described in this paper is version JFJX01000000. The raw Illumina reads are available at ENA/SRA under accession number PRJEB5959 (ERP005406).
